# Association of 3p27.1 Variants with Whole Body Lean Mass Identified by a Genome-wide Association Study

**DOI:** 10.1038/s41598-020-61272-z

**Published:** 2020-03-09

**Authors:** Shu Ran, Yu-Xue Zhang, Lu Liu, Zi-Xuan Jiang, Xiao He, Yu Liu, Hui Shen, Qing Tian, Yu-Fang Pei, Hong-Wen Deng, Lei Zhang

**Affiliations:** 1 School of Medical Instruments and Food Engineering, University of Shanghai for Science and Technology, Shanghai, PR China; 2 Kunshan Hospital of Traditional Chinese Medicine, Jiangsu, PR China; 3 Department of Biostatistics and Bioinformatics, Tulane University School of Public Health and Tropical Medicine, New Orleans, Louisiana USA; 4 Department of Epidemiology and Statistics, School of Public Health, Soochow University, Jiangsu, PR China; 5 Jiangsu Key Laboratory of Preventive and Translational Medicine for Geriatric Diseases, Soochow University, Jiangsu, PR China; 6 Center for Genetic Epidemiology and Genomics, School of Public Health, Soochow University, Jiangsu, PR China

**Keywords:** Genome-wide association studies, Genome-wide association studies, Risk factors, Risk factors

## Abstract

Whole body lean mass (WBLM) is a heritable trait predicting sarcopenia. To identify genomic locus underlying WBLM, we performed a genome-wide association study of fat-adjusted WBLM in the Framingham Heart Study (FHS, N = 6,004), and replicated in the Kansas City Osteoporosis Study (KCOS, N = 2,207). We identified a novel locus 3p27.1 that was associated with WBLM (lead SNP rs3732593 P = 7.19 × 10^−8^) in the discovery FHS sample, and the lead SNP was successfully replicated in the KCOS sample (one-sided P = 0.04). Bioinformatics analysis found that this SNP and its adjacent SNPs had the function of regulating enhancer activity in skeletal muscle myoblasts cells, further confirming the regulation of WBLM by this locus. Our finding provides new insight into the genetics of WBLM and enhance our understanding of sarcopenia.

## Introduction

Sarcopenia is defined as a progressive, and generalized loss of skeletal muscle mass, strength and function^1^. Sarcopenia plays a vital role in the frailty process, also being a key player in its incubation period and it causes serious consequences through frailty, such as decreased function, disability and eventual death^2,3^. According to Khosla *et al*.^4^, the age- and sex-adjusted prevalence of sarcopenia varied from 6 to 15% among subjects 65 years of age or over. The muscular tissue, as characterized by Lean Body Mass (LBM), is frequently used to predict sarcopenia. LBM can be measured accurately by dual energy X-ray absorptiometry (DXA).

Previous studies showed that LBM is under genetic control, with heritability over 50%^5,6^. Previous studies have identified dozens of genomic loci associated with LBM^7–11^. Among them, Zillikens *et al*.^12^ identified five loci in/near *HSD17B11*, *VCAN*, *ADAMTSL3*, *IRS1*, and *FTO* that were associated with lean body mass. Medina-Gomez *et al*.^13^ conducted a bivariate genome-wide association meta-analysis of pediatric musculoskeletal traits and revealed pleiotropic effects at the *SREBF1*/*TOM1L2* locus. However, to date, the identified loci only explain a small proportion of the variation observed for a particular phenotype, and the majority of hidden heritability is yet to be identified.

In this study, we performed a GWAS of WBLM using the Framingham Heart Study (FHS) as discovery sample and the Kansas City Osteoporosis Study (KCOS) as replication sample. In addition, we conducted a serious of bioinformatic analysis to explore the functional relevance of the identified variants.

## Materials and Methods

All the methods were conducted in accordance with the guidelines and regulations of the Soochow university institutional review board (for the Framingham heart study sample) and the University of Missouri Kansas City institutional review board (for the Kansas City osteoporosis study). The Institutional Review Boards of University of Missouri Kansas City and the Soochow university approved the study. All participants signed informed consent before participating.

### Discovery sample

The Framingham heart study (FHS) was accessed through the database of genotype and phenotype (dbGAP) portal. The FHS sample is a longitudinal and prospective cohort consisting of over 16,000 individuals spanning three generations of European ancestry. All participants underwent dual-energy X-ray absorptiometry (DXA, Lunar Corp., Madison, WI, USA) scan during different examinations. More details about the FHS sample have been described elsewhere^14^.

A subset sample of the FHS cohort was genotyped by the Affymetrix high-throughput 500 K genotyping array plus a supplemental 50 K genotyping array. These two genotype sets were merged together to form a single dataset of ~550,000 SNPs.

After checking the availability of both genotypes and phenotypes, we identified 6,004 Caucasian subjects aged 23–93 years, of which 637, 2,222, and 3,145 were from the original, offspring, and third generations, respectively.

### Replication sample

The replication sample was the Kansas City Osteoporosis Study (KCOS). The KCOS is a cross-sectional study of osteoporosis with 2,286 unrelated European ancestry participants living in and around Kansas City, Missouri, USA. Participants are normal healthy subjects defined by a complete set of exclusion criteria, as described elsewhere^15^. The KCOS cohort was genotyped by the Affymetrix SNP 6.0 genotyping array. WBLM was measured by a DXA bone densitometer (QDR 4500 W, Hologic Inc., Bedford, MA, USA) according to the manufacture protocol.

After checking the availability of both genotypes and phenotypes, we identified 2,207 individuals aged 18–92 years.

### Phenotype modeling

In both discovery and replication samples, the stepwise linear regression model implemented in the R function stepAIC was used to screen the significance of covariates, including whole body fat mass, gender, age, age^^2^, height, height^^2^ and the first five principle components derived from genome-wide genotype data. Raw WBLM was adjusted by significant covariates, and the residuals were normalized by inverse quantiles of standard normal distribution.

### Genotype quality control

We followed strict genotype quality control (QC) procedure at both individual and SNP levels. At the individual level, genetic sex was inferred from genotype data on X-chromosome with PLINK^16^ and was compared with the self-reported sex. Individuals of inconsistent sex were removed. At the SNP level, SNP that violates the Hardy-Weinberg equilibrium (P < 1.0 × 10^−5^) were removed. Population outliers were monitored by genotype-derived principle components, and if present, outliers were removed. In the FHS sample, SNPs with the Mendel error were set to missing value.

### Genotype imputation

Both discovery and replication samples were imputed using sequencing data generated by the 1000 genomes project^17^. Specifically, phased variants of 240 individuals of European ancestry were downloaded from the project website. Haplotypes of bi-allelic variants, including SNPs and bi-allelic insertions/deletions (INDELs), were extracted to form reference panels for imputation. As a QC procedure, variants with zero or one copy of minor alleles were removed.

Before imputation, it is necessary to check for the consistency of allele strandedness between the test sample and the reference sample. The chi-square test was used to examine the consistency. SNPs that failed the consistency test (p < 1.0 × 10^−6^) were transformed into the reverse strand in the test sample. SNPs that again failed the consistency test were removed. Imputation was performed with FISH^18^, a fast and accurate diploid genotype imputation algorithm.

The imputation certainty was measured by the imputation score r^2^, which was defined as the correlation between imputed dosage and the best imputed genotype. Variants of low imputation score (r^2^ < 0.3) or of low frequency (MAF < 0.05) were excluded from subsequent analyses.

### Association testing

In the FHS sample, an additive mode of inheritance was applied to test genetic association between normalized phenotype residuals with genotyped and imputed genotypes. A mixed linear regression model was applied to account for genetic relatedness within each pedigree in the FHS sample^19^. Association test was examined within the variance-components framework.

Association was examined in the KCOS sample by a linear regression model with MACH2QTL^20^.

### Functional annotation

Functional annotation of the identified SNPs was performed using the bioinformatical software HaploReg^21^. HaploReg provides functional information for non-coding SNPs with multiple functional categories, including conservation sites, DNase hypersensitivity sites (DHS), transcription factor binding sites (TFBS), promoter sites, enhancer sites, and others. We annotated lead SNP and its neighbor SNPs with strong LD pattern (r^2^ > 0.8).

We used the GTEx project dataset^22^ and the Westra *et al*.‘s study^23^ to perform cis-eQTL analysis. The GTEx project collects and analyzes a variety of human tissues from donors of the same dense genotype to assess genetic variation within their genome. We downloaded the summary results of skeletal muscle tissue from the GTEx website (V7) (https://www.gtexportal.org/home/). The Westra *et al*.‘s study performed eQTL meta-analysis reported in non-transformed peripheral blood samples of 5,311 individuals, with replication in 2,775 individuals^23^. We obtained cis-eQTL results from the study website (http://www.genenetwork.nl/bloodeqtlbrowser/).

### Replication of previously identified loci

We assessed the GWAS Catalog (https://www.ebi.ac.uk/gwas/) web portal to identify loci that were previously reported for lean mass. For each locus, we listed effect size and p-value of the lead SNP in the present study to evaluate the replicability of those previously reported loci.

### Ethics approval and consent to participate

The study was approved by the local institutional review board of all agencies. All participants signed informed consent before participating in the study.

## Results

Basic characteristics of both discovery and replication samples are listed in Table 1. A total of 6,004 subjects are available in the FHS sample for analysis, including 637, 2,222 and 3145 from the first, offspring and third generation, respectively, 3,479 of whom are women. In the KCOS sample, a total of 2,207 subjects are available for analysis, 1,691 of whom are women.

**Table 1:** Basic characteristics of discovery sample and replication sample.

	Discovery sample (FHS)		Replication sample (KCOS)	
	Male	Female	Male	Female
No. of subjects	2,525	3,479	516	1,691
Age	53.98(13.13)	55.90(13.67)	51.22(16.14)	51.65(12.89)
Height (cm)	175.98(7.11)	161.98(6.81)	175.85(7.31)	163.29(6.26)
Weight (kg)	84.42(13.32)	67.95(13.77)	86.79(16.27)	71.44(16.00)
Whole body fat mass (kg)	24.93(8.99)	27.78(10.45)	20.62(9.07)	25.28(10.75)
Whole body lean mass (kg)	57.29(7.15)	38.27(5.23)	66.34(9.45)	46.82(7.04)

Note: The numbers within parentheses are standard deviation (SD).

**Figure 1:**
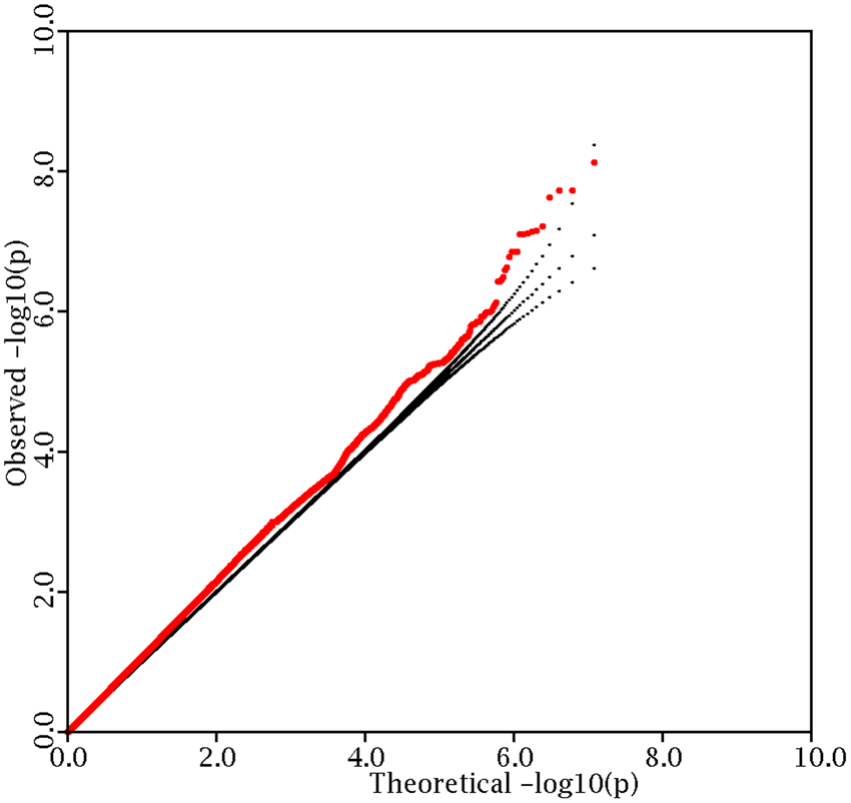
QQ plot. Results were plotted for fat-adjusted whole body lean mass in the Framingham heart study.

In the FHS sample, after performing genotype imputation and removing variants with low frequency and poor imputation certainty, a total of 6,879,267 variants were available for analysis, of which 88% were SNPs and the rest were bi-allelic deletion/insertion variants (DIVs). Overall genomic control inflation factor was 1.09. The logarithmic quantile–quantile (QQ) plot results is displayed in Fig. 1. The QQ plot shows a significant deviation in the distribution tail, implying the presence of a true association signals.

**Figure 2:**
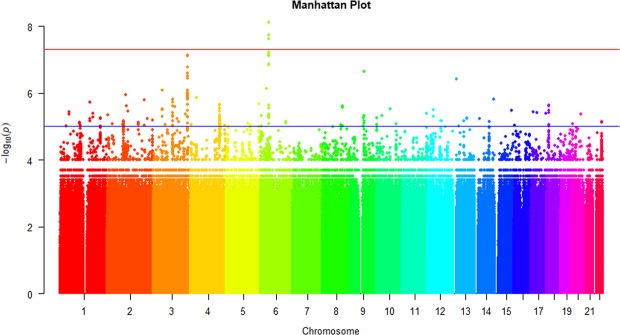
WBLM genome-wide association manhattan plot.

Manhattan plot of association results across the genome is displayed in Fig. 2. At the genome-wide significance (GWS, 5.0 × 10^−8^) level, a total of 4 SNPs at one single locus 6p21.1 were identified, with the lead SNP rs513688 (P = 7.48 × 10^−9^). In a previous study, this locus was found to be associated with leg lean mass^24^ with the same lead SNP, indicating that they arise from one single association signal.

SNPs from one additional locus 3p27.1 showed evidence of association. The lead SNP rs3732583 at this locus nearly reaches the GWS level (P = 7.19 × 10^−8^). The main association results are listed in Table 2.

**Table 2:** Main association results of whole body lean mass at 3p27.1.

rs#	Position	Alleles	EAF	FHS (N = 6004)			KCOS (N = 2207)		
				Beta	SE	P	Beta	SE	P (one-sided)
rs3732593	183036166	G/T	0.77	0.12	0.02	7.19 × 10^−8^	0.07	0.04	0.04
rs13434301	183036526	C/T	0.23	−0.12	0.02	7.80 × 10^−8^	−0.07	0.04	0.05

Notes: Physical position is based on the human genome GRCH37 assembly. The first and second alleles represent the effect and alternative alleles. EAF is the effect allele frequency. Beta is regression coefficient of the effect allele.

In the replication sample, rs3732593 was consistent in effect direction to that in the discovery sample. Further, the replication association is nominal significant (one-sided P = 0.04), further supporting the association at this locus.

**Figure 3:**
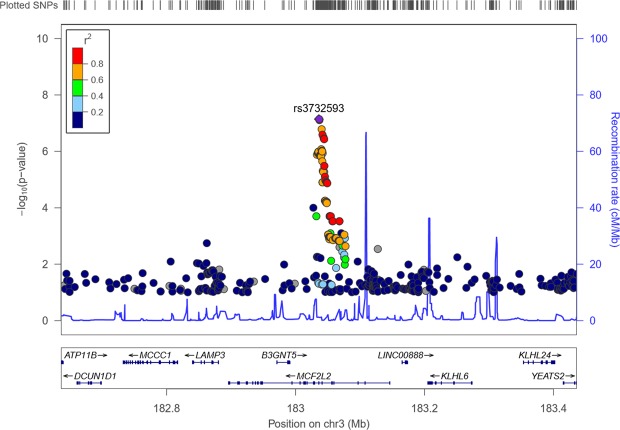
Regional plot of the associated region 3p27.1.

Regional plot of the lead SNP rs3732593 for the novel locus 3p27.1 is displayed in Fig. 3.

### Functional annotation

We used HaploReg to annotate the lead SNP rs3732593 at 3p27.1 and its neighboring SNPs (LD r^2^ > 0.8). rs3732593 is located in the intron of MCF2 cell line derived transforming sequence-like 2 (*MCF2L2*) gene. In the latest GTEx cis-eQTL summary results (version 7), rs3732593 is associated with the expression of ATPase phospholipid transporting 11B (*ATP11B*, P = 0.01) gene in skeletal muscle tissue. In the Roadmap epigenomic study, it is predicted to have enhancer site activity in skeletal myoblasts cells, as implied by core 15-state model and H3K4me1 histone mark. It has 23 neighboring SNPs in strong LD (r^2^ > 0.8); all are located in intron of *MCF2L2*. Three of them (rs6767909, rs6770912 and rs12630205, all within a 12.5 kb window) are associated with the expression of *MCF2L2* in the study of Westra *et al*. in whole blood tissue. They are also associated with the expression of another gene betaGal beta-1,3-N-acetylglucosaminyltransferase 5 (*B3GNT5*) in the same study.

### Replication of previously reported loci

The previous studies identified 22 loci for lean mass traits; none of them are significant at the GWS level in the present study. This is probably because of the limited sample size used in the present study.

At the nominal level (P < 0.05), on the other hand, the present study did replicate a total of 4 previously identified loci. Among them, 3 loci are further consistent in effect direction, including 2q36.3 (lead SNP rs2943656, beta = −0.06, p = 2.0 × 10^−3^), 16q12.2 (rs9936385, beta=0.06, p = 2.4 × 10^−3^) and 1p36.12 (rs6684375, beta= 0.05, p = 4.9 × 10^−2^).

## Discussion

In this study, we performed a genome-wide association study of fat adjusted whole body lean mass in 6,004 participants from the FHS, and performed replication in the KCOS. we identified one novel locus 3p27.1 that was associated with WBLM after adjustment by whole body fat mass (lead SNP rs3732593 P = 7.19 × 10^−8^).

Previous study suggested that the third decade is a turning point in muscle mass and the age of 27 years as the threshold, beyond which skeletal mass began to be inversely related to the age of men and women^25^. Other studies showed that men’s lean body mass and skeletal muscle mass were significantly higher than women’s^4,26^. In our study, the age and gender were included as covariates. In addition, age square was included as covariate too, to account for the non-linear influence of age to lean mass (e.g., increase to the peak, then decrease).

Moreover, both fat mass and lean mass are important body composition and they are highly correlated. In this study, because of the consistent correlation between fat and lean mass, we chose whole body fat mass as one of the covariates for adjusting whole body lean mass to eliminate the effects of confounding factors. This allows us to estimate the independent effects of lean mass.

Functional annotations highlighted several candidate genes, including *MCF2L2*, *ATP11B* and *B3GNT5*. Among them, *ATP11B* is phosphorylated in its intermediate state and drives uphill transport of ions across membranes, which are members of the P-type ATPase. P-type ATPases are a family of ATP-dependent ion transporters. The genes most closely related to *ATP11B* are ATPase phospholipid transporting 11 A (*ATP11A*) and the type IIB sarco/endoplasmic reticulum Ca^2+^ transporter (*SERCA1*)^27^. SERCA Ca^2+^-ATPases is intracellular pumps located in the sarcoplasmic or endoplasmic reticula of muscle cells^28,29^. It is involved in muscle stimulation and contraction. Mutations in gene *SERCA1* cause changes in muscle properties and dysfunction-related diseases. However, the functions of *ATP11B* gene related to skeletal muscle is still unknown.

*MCF2L2* gene encodes a guanine nucleotide exchange factor of the Rho family, which plays an important role in the signaling pathway of the Rho protein. Studies have shown that the Rho-guanine nucleotide exchange factor domain of Obscurin can activate RhoA signaling in skeletal muscle^30^. As a small GTPase of the rho family, RhoA has been well documented to regulate actin reorganization, regulate transcription and participate in cell cycle control^31,32^. It plays a key role in the development and maintenance of skeletal muscle.

Meta-analysis of multiple individual studies has now become the mainstream of GWAS analysis. The biggest advantage of meta-analysis is to enhance the statistical power of identifying true genetic associations by expanding the sample size. However, modern meta-analysis introduces heterogeneity problems because each sample study was not designed under the same conditions. Although individual studies comprising smaller sample sizes than typical meta-analyses, they are more uniform in terms of sample and experimental design than the latter. In addition, both the discovery and replication samples in this study represented populations of European ancestry. This eliminates the erroneous correlation signals that may result from mismatched ancestors between samples. Therefore, there is irreplaceable value in individual research.

There were 2 different DXA machines used in the discovery and the replication samples, respectively. However, only one machine was used within each sample, and association analysis was done within each sample. As we did not combine raw phenotypes of the two samples, no systematic bias due to machine type would generate. Within each sample, the same phenotype modeling approach was used. The phenotype being analyzed in both samples follows strictly a standard normal distribution so that the regression coefficient was comparable between both samples.

The identified lead SNP rs3732593 was extremely significant (P = 7.19 × 10^−8^) in the discovery FHS sample but was only nominally significant (P = 0.04) in the replication sample. Due to the winner’s curse effect, replication p-value is difficult to be as low as that in the discovery sample even for true association^33^. Instead, a nominally significant replication p-value would suggest a successful replication. In addition to p-value, we also checked the effect direction across the discovery and the replication samples. The consistent effect direction strenghtened our confidence towards true association at this locus.

In conclusion, by performing a genome-wide association study and related functional analysis, we identified a novel locus 3p27.1 that is associated with whole body lean mass. Our findings provide useful insights that enhance our understanding of the genetic pathogenesis of sarcopenia.

## Data availability

Summary results are available upon request to the corresponding author.
